# Prediction of potential prognostic biomarkers in metastatic prostate cancer based on a circular RNA-mediated competing endogenous RNA regulatory network

**DOI:** 10.1371/journal.pone.0260983

**Published:** 2021-12-03

**Authors:** Liang Luo, Lei-Lei Zhang, Wen Tao, Tao-Lin Xia, Liao-Yuan Li

**Affiliations:** 1 Department of Urology, The Third Affiliated Hospital, Sun Yat-sen University, Guangzhou, China; 2 Department of Urology, Foshan First Municipal People’s Hospital, Foshan, China; Sun Yat-sen University, CHINA

## Abstract

Recently, studies on competing endogenous RNA (ceRNA) networks have become prevalent, and circular RNAs (circRNAs) have crucial implications for the development and progression of carcinoma. However, studies relevant to metastatic prostate cancer (mPCa) are scant. This study aims to discover potential ceRNAs that may be related to the prognosis of mPCa. RNA-Seq data were obtained from the MiOncoCirc database and Gene Expression Omnibus (GEO). Differential expression patterns of RNAs were examined using R packages. Circular RNA Interactome, miRTarBase, miRDB and TargetScan were applied to predict the corresponding relation between circRNAs, miRNAs and mRNAs. The Gene Ontology (GO) annotations were performed to present related GO terms, and Gene Set Enrichment Analysis (GSEA) tools were applied for pathway annotations. Moreover, survival analysis was conducted for the hub genes. We found 820 circRNAs, 81 miRNAs and 179 mRNAs that were distinguishingly expressed between primary prostate cancer (PCa) and mPCa samples. A ceRNA network including 45 circRNAs, 24 miRNAs and 56 mRNAs was constructed. In addition, the protein–protein interaction (PPI) network was built, and 10 hub genes were selected by using the CytoHubba application. Among the 10 hub genes, survival analysis showed that ITGA1, LMOD1, MYH11, MYLK, SORBS1 and TGFBR3 were significantly connected with disease-free survival (DFS). The circRNA-mediated ceRNA network provides potential prognostic biomarkers for metastatic prostate cancer.

## Introduction

In men, prostate cancer (PCa) is a type of malignant tumor of the urogenital system with high morbidity. Patients with de novo metastatic PCa (mPCa) have a high risk of disease‐specific mortality [1]. Previous studies have reported that prostate-specific antigen (PSA) testing can help reduce the number of patients with metastatic cancer. Despite screening for earlier diagnosis, many men develop metastases [2]. Many studies have reported that ceRNA networks are crucial for PCa biology and therapeutics [3, 4]. Nevertheless, there is insufficient understanding of the molecular mechanism that may cause mPCa. Accordingly, it is essential to explore effective biomarkers that can improve the perception of the development and progression of mPCa.

CircRNAs are noncoding RNAs from a noncanonical splicing event called backsplicing, and their covalently closed ring structure prevents them from exonuclease-mediated degradation, which results in high stability [5]. A relevant study showed that circRNAs acted as miRNA sponges, functioned through interactions with proteins, and participated in the protein synthesis [4]. Accumulating evidence suggests that circRNAs impact physiological development and multifarious diseases such as brain development and diseases, cardiovascular diseases and cancers [6].

A ceRNA hypothesis was raised by Pandolfi et al., which indicates that all types of RNA transcripts harboring miRNA response elements (MREs) could serve as one type of ceRNA, which may influence the tumorigenesis of malignant tumors, such as mRNAs, pseudogenes, circRNAs and lncRNAs [7]. Certain studies have shown that ceRNA interactions have implications for gastric cancer, mammary cancer, melanoma, spongioblastoma, prostate cancer, etc [8].

**Fig 1:**
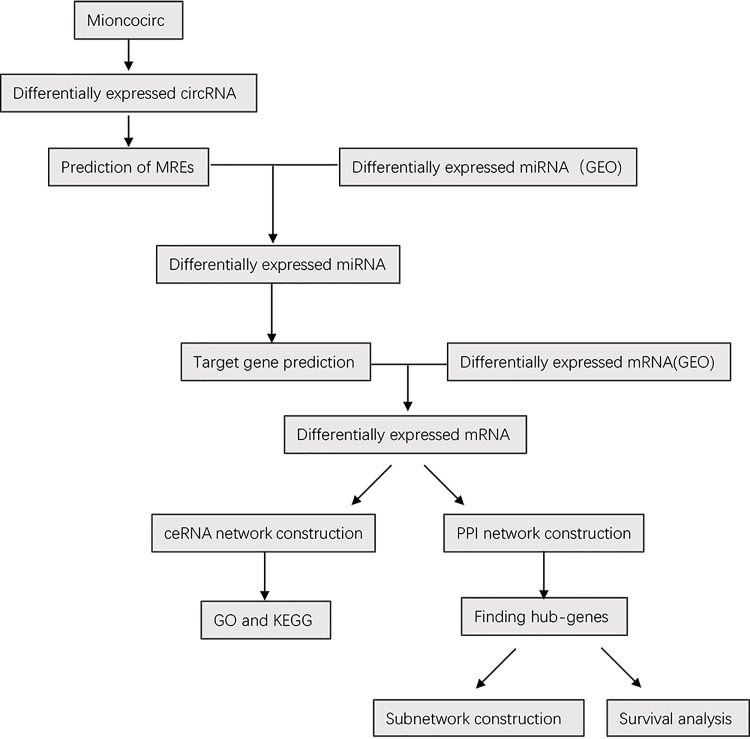
Flow diagram of the ceRNA analysis.

In this study, RNA-seq data were obtained from the MiOncoCirc and GEO databases. We identified the circRNAs that acted as miRNA sponges and found target genes for miRNAs. Next, a ceRNA network was built, including 45 circRNAs, 24 miRNAs and 56 mRNAs. GO and GSEA were applied for differentially expressed mRNAs. Finally, we established a PPI network to better comprehend the molecular mechanism and pathogenesis of mPCa. This study aims to determine the relationships between ceRNAs and the carcinogenesis and progression of mPCa and provide further insight into the improvement of the treatment and prognosis of mPCa. The flow diagram of the entire ceRNA analysis is presented in Fig 1.

## Materials and methods

### MiOncoCirc compendium

The MiOncoCirc compendium is an accessible compendium of cancer-focused circRNAs for the scientific community [9]. In this study, the circRNA profile data of 88 mPCa patients were retrieved from MiOncoCirc (https://MiOncoCirc.github.io/). A complete description of the donor age range, sample location and cell lines was obtained from relevant annotation documents.

### GEO database

The circRNA data of 144 primary PCa tissues were downloaded from the GSE113120 dataset of the GEO database (http://www.ncbi.nlm.nih.gov/geo). The expression patterns of miRNA and mRNA were acquired from GSE21036 (14 mPCa tissues and 99 primary PCa tissues) and GSE21034 (19 mPCa tissues and 131 primary PCa tissues), respectively. Annotation information on the platform was used to convert probes to the corresponding gene symbols.

### Screening of differentially expressed genes (DEGs)

Limmapackage of R (v3.6.1) was employed to identify DEGs between mPCa tissues and primary PCa tissues, and the differences were described by fold-change (FC) and false discovery rate (FDR). |Log2FC|>5 and FDR<0.05 were considered significant for circRNAs. The differential expression levels of mRNAs and miRNAs were analyzed with cutoff values of |Log2FC|>1 and FDR<0.05. Then, we used pheatmap packages to draw heatmaps for the obtained DEGs.

### Construction of ceRNA network

The Circular RNA Interactome (https://circinteractome.nia.nih.gov/) was applied to predict the circRNA-miRNA interactions. Then, the MiRDB (http://www.mirdb.org/), miRTarBase (http://mirtarbase.mbc.nctu.edu.tw/) and TargetScan (http://www.targetscan.org/) databases were employed to explore the corresponding mRNAs for miRNAs. Finally, a ceRNA network was constructed by selected circRNAs, miRNAs and mRNAs and visualized by Cytoscape (v3.7.2; https://cytoscape.org/).

### Protein–protein interaction (PPI) network construction

We employed the String database (https://string-db.org/) to analyze the interactions between proteins of DEmRNAs. Then, the network was visualized by using Cytoscape. The CytoHubba tool was used to obtain 10 hub genes ranked by degree.

### Gene Ontology (GO) and Kyoto Encyclopedia of Genes and Genomes (KEGG) analysis

The ClusterProfiler package of R was employed to identify the GO terms. GO consists of three gene aspects: biological process, cellular component and molecular function. GSEA (https://www.gsea-msigdb.org/) was applied to analyze the KEGG enrichment analysis. P<0.05 was considered statistically significant.

### Survival analysis

To identify specific correlations of the expression levels of 10 hub genes with patient prognosis, Gene Expression Profiling Interactive Analysis (GEPIA, http://gepia.cancer-pku.cn/) was applied for a survival analysis. We set the median as the group cutoff, calculated the hazards ratio based on the Cox PH Model, and added the 95% confidence interval as a dotted line. Finally, the corresponding disease-free survival (DFS) was obtained. The threshold for survival prognosis significance was P< 0.05.

## Results

### Identification of differentially expressed circRNAs (DEcircRNAs)

**Fig 2:**
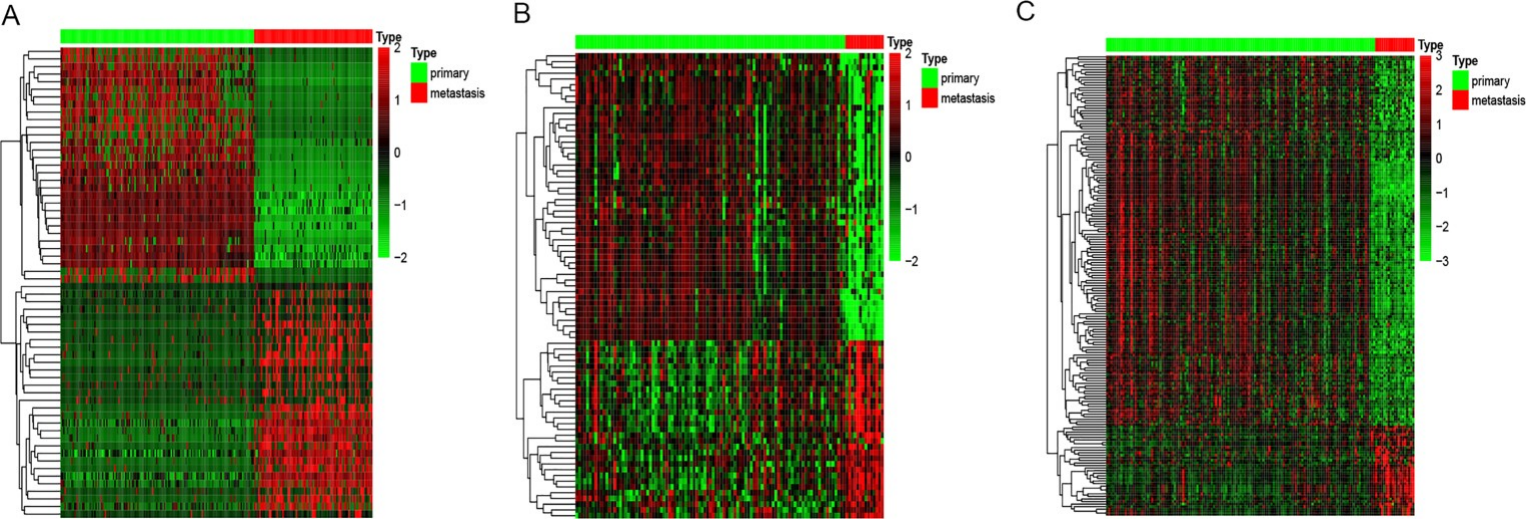
(A) Heatmaps of DEcirRNAs between primary prostate cancer (PCa) and metastatic prostate cancer (mPCa) of MiOncoCirc and GEO. (B) Heatmaps of DEmiRNAs between PCa and mPCa of GEO. (C) Heatmaps of DEmRNAs between PCa and mPCa of GEO.

We examined the expression level of circRNAs between 144 primary PCa and 88 mPCa samples. Setting |Log2FC|>5 and FDR<0.05 as the cutoffs, we obtained 31 significantly upregulated and 31 significantly downregulated circRNAs (Fig 2A; Table 1).

**Table 1:** Top 10 differential circRNAs for prostate cancer.

circRNAs	logFC	P Value	FDR
hsa_circ_0027821	-7.602252641	6.14E-39	1.67E-35
hsa_circ_0059780	-5.002149971	2.39E-37	8.67E-35
hsa_circ_0075897	9.149428043	2.57E-37	8.67E-35
hsa_circ_0046862	-5.346407854	2.87E-37	8.67E-35
hsa_circ_0054225	-5.043921793	8.48E-38	8.67E-35
hsa_circ_0011178	-8.117888361	2.65E-37	8.67E-35
hsa_circ_0012843	5.47286257	4.81E-37	1.06E-34
hsa_circ_0006309	-5.038449863	1.05E-36	1.68E-34
hsa_circ_0086324	6.160337638	1.87E-36	2.54E-34
hsa_circ_0019868	6.080732178	4.23E-36	5.47E-34

### Identification of differentially expressed miRNAs (DEmiRNAs) and mRNAs (DEmRNAs)

Subsequently, 340 miRNAs and 16045 mRNAs were separately explored. |Log2FC|>1 and FDR<0.05 were used as cutoffs, and we identified 81 DEmiRNAs (Fig 2B; Table 2), including 31 upregulated and 50 downregulated miRNAs, and 179 DEmRNAs (Fig 2C; Table 3), including 35 upregulated and 144 downregulated mRNAs.

**Table 2:** Top 10 differential miRNAs for prostate cancer.

miRNAs	logFC	P Value	FDR
hsa-miR-1	-5.445933682	1.77E-09	2.30E-07
hsa-miR-143	-4.041918181	2.44E-09	2.30E-07
hsa-miR-145	-3.215684161	2.72E-09	2.30E-07
hsa-miR-133a	-3.012035522	9.54E-09	5.39E-07
hsa-miR-133b	-4.376786098	1.30E-08	6.28E-07
hsa-miR-23b	-1.945219895	3.55E-08	1.20E-06
hsa-miR-24-1	-1.740426749	3.06E-08	1.20E-06
hsa-miR-27b	-1.755807868	3.37E-08	1.20E-06
hsa-miR-221	-2.406665523	4.54E-08	1.40E-06
hsa-miR-130a	-1.964884765	1.38E-07	3.89E-06

**Table 3:** Top 10 differential mRNAs for prostate cancer.

mRNAs	logFC	P Value	FDR
ACTG2	-4.067886307	2.25E-12	5.67E-09
CHRDL1	-2.398511617	3.95E-12	5.67E-09
CNN1	-2.892090904	2.16E-12	5.67E-09
CNTN1	-1.896213621	4.46E-12	5.67E-09
DES	-1.850000006	3.80E-12	5.67E-09
LMOD1	-1.644128713	3.95E-12	5.67E-09
MEIS2	-2.034462529	5.44E-12	5.67E-09
MYH11	-3.886474917	2.44E-12	5.67E-09
MYL9	-1.972759519	3.37E-12	5.67E-09
PCP4	-2.437681131	4.83E-12	5.67E-09

### Construction of a circRNA-miRNA-mRNA network

**Fig 3:**
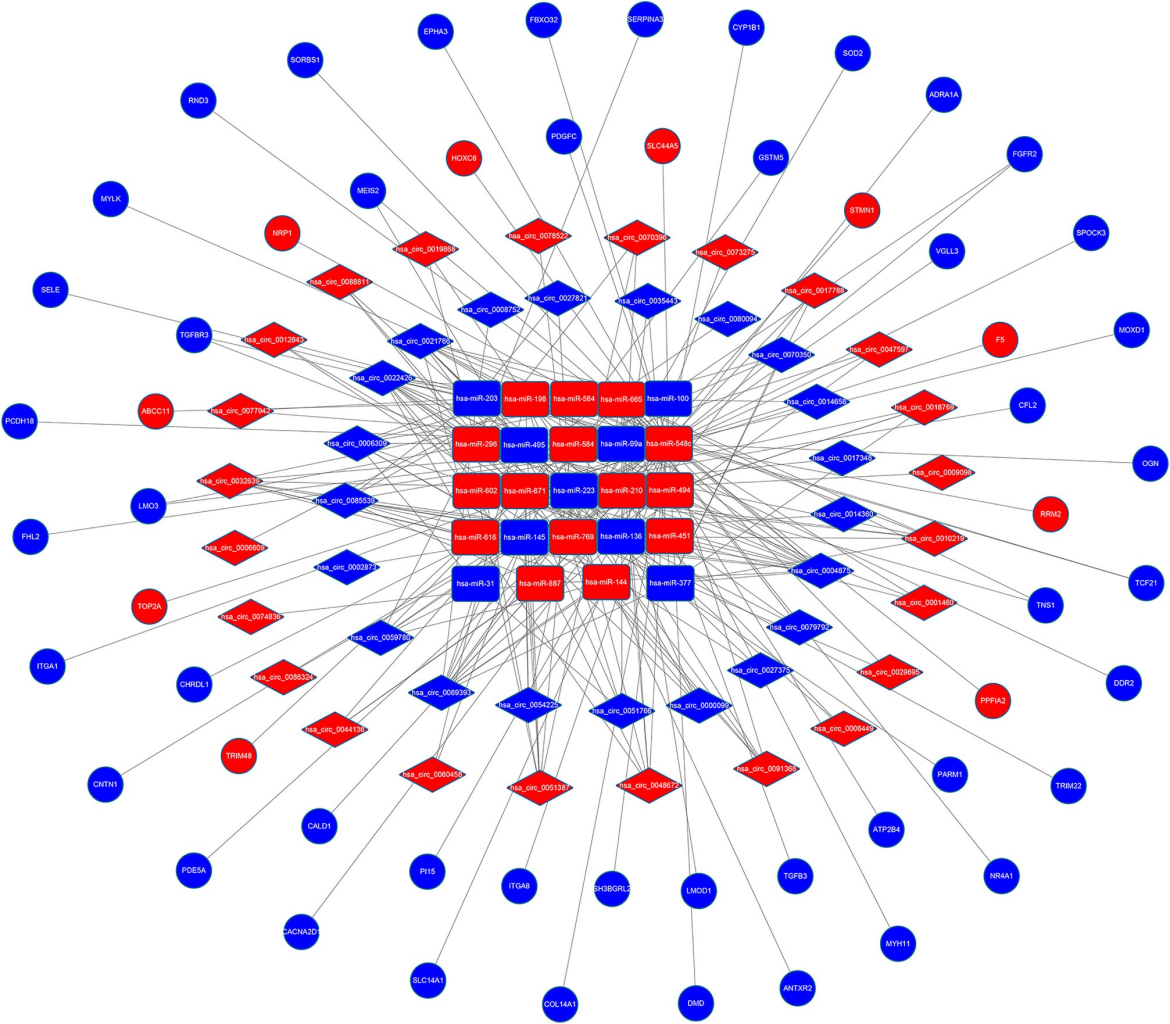
The circRNA-miRNA-mRNA ceRNA network in mPCa. The network comprises 45 circRNA nodes, 24miRNA nodes and 56mRNA nodes. The rectangles indicate miRNAs, the diamonds indicate circRNAs, and the ellipses indicate mRNAs. The red nodes represent increased expression, and the blue nodes represent decreased expression.

Depending on the CircInteractome online database, we explored the corresponding miRNAs of DEcircRNAs. After intersecting with DEmiRNAs, 45 of 62 DEcircRNAs were found to target 24 of 81 DEmiRNAs. Then, miRDB, miRTarBase and TargetScan were adapted to predict the target mRNAs of 24 circRNA-targeted miRNAs, and only mRNAs identified by at least two of these databases were selected. The selected target mRNAs then overlapped with DEmRNAs. Finally, we obtained a ceRNA regulatory network constructed of 45 circRNAs, 24 miRNAs and 56 mRNAs and visualized it by using Cytoscape v3.7.2 (Fig 3).

### PPI network construction

**Fig 4:**
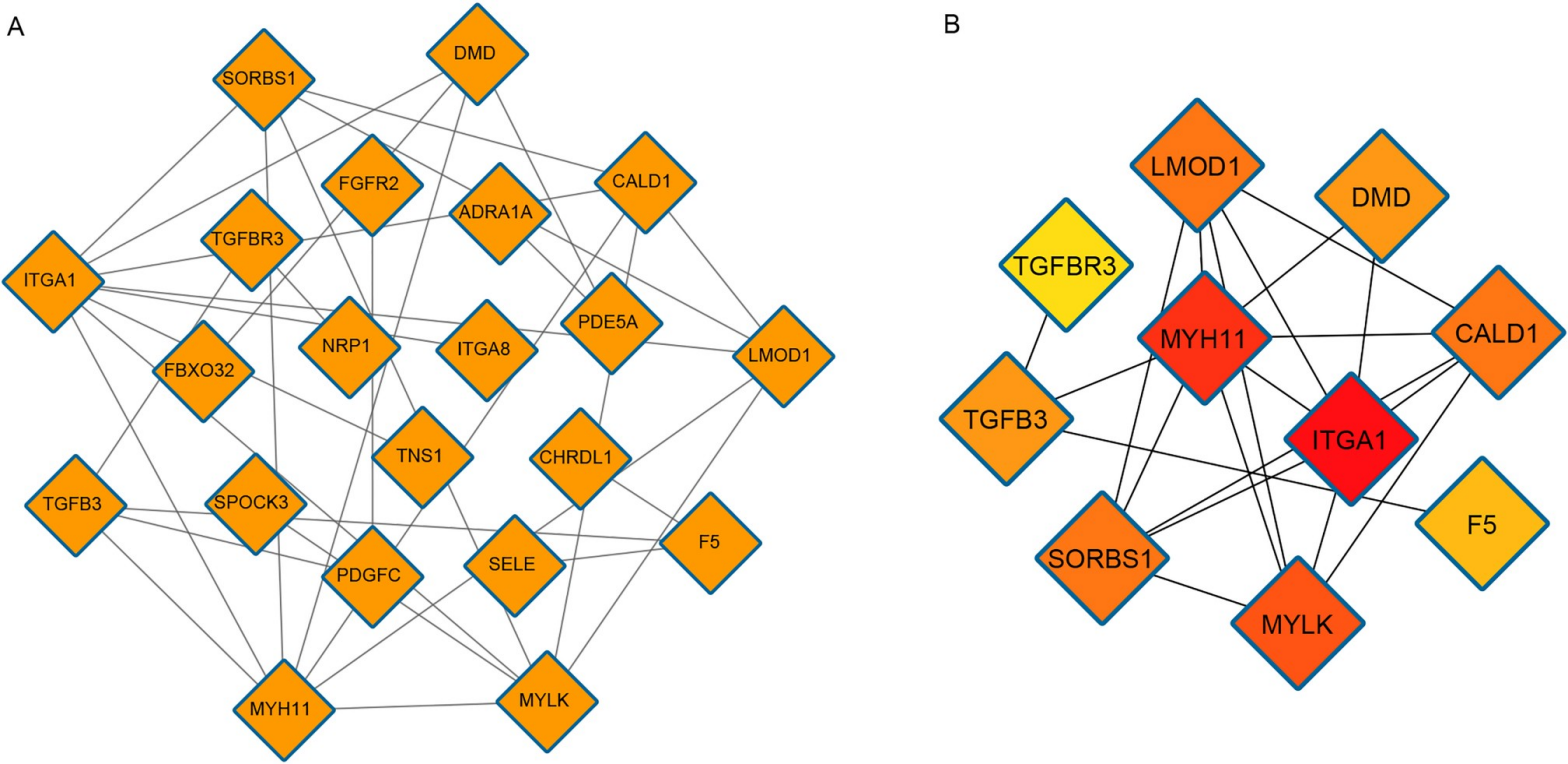
PPI network and corresponding hub genes. (A) The network includes 21 nodes and 31 edges. (B) 10 hub genes. The node color gradually changes from yellow to red, which indicates the ascending order of the degree of the genes.

**Fig 5:**
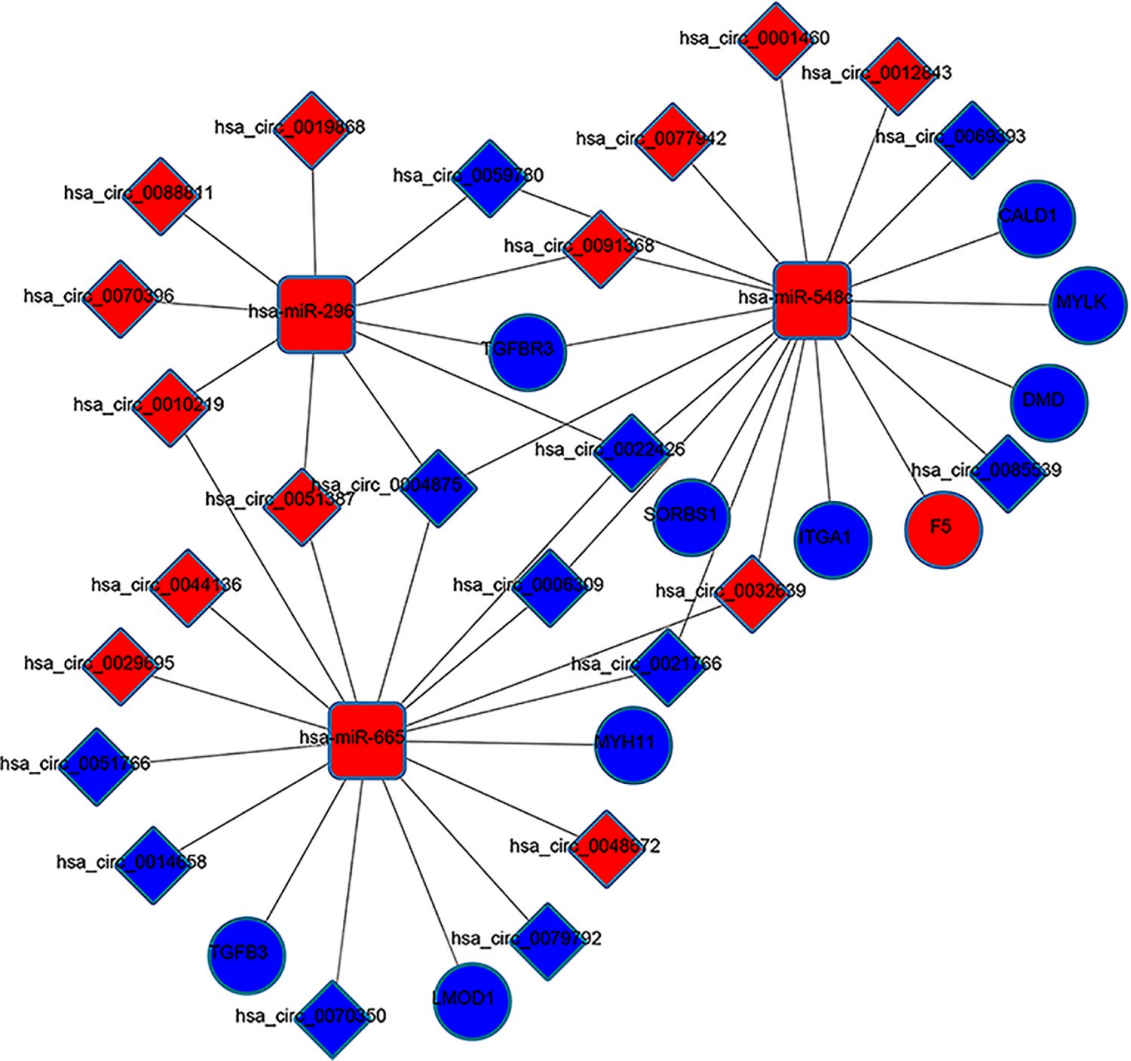
circRNA-miRNA-hub gene subnetwork. The network consists of 24circRNAs, 3 miRNAs and 10 mRNAs. The rectangles indicate miRNAs, the diamonds indicate circRNAs, and the ellipses indicate mRNAs. The red nodes represent increased expression, and the blue nodes represent decreased expression.

We identified the mutual effect between proteins of DEmRNAs through the STRING online database and constructed a PPI network including 21 nodes and 31 edges (Fig 4A). Then, we evaluated the degree between proteins to find the hub genes (Fig 4B). Based on the rank method of CytoHubba, we obtained 10 hub genes: ITGA1, MYH11, MYLK, SORBS1, CALD1, LMOD1, TGFB3, DMD, F5, and TGFBR3. Subsequently, a circRNA-miRNA-hub gene subnetwork was built (Fig 5).

### Functional assessment

**Fig 6:**
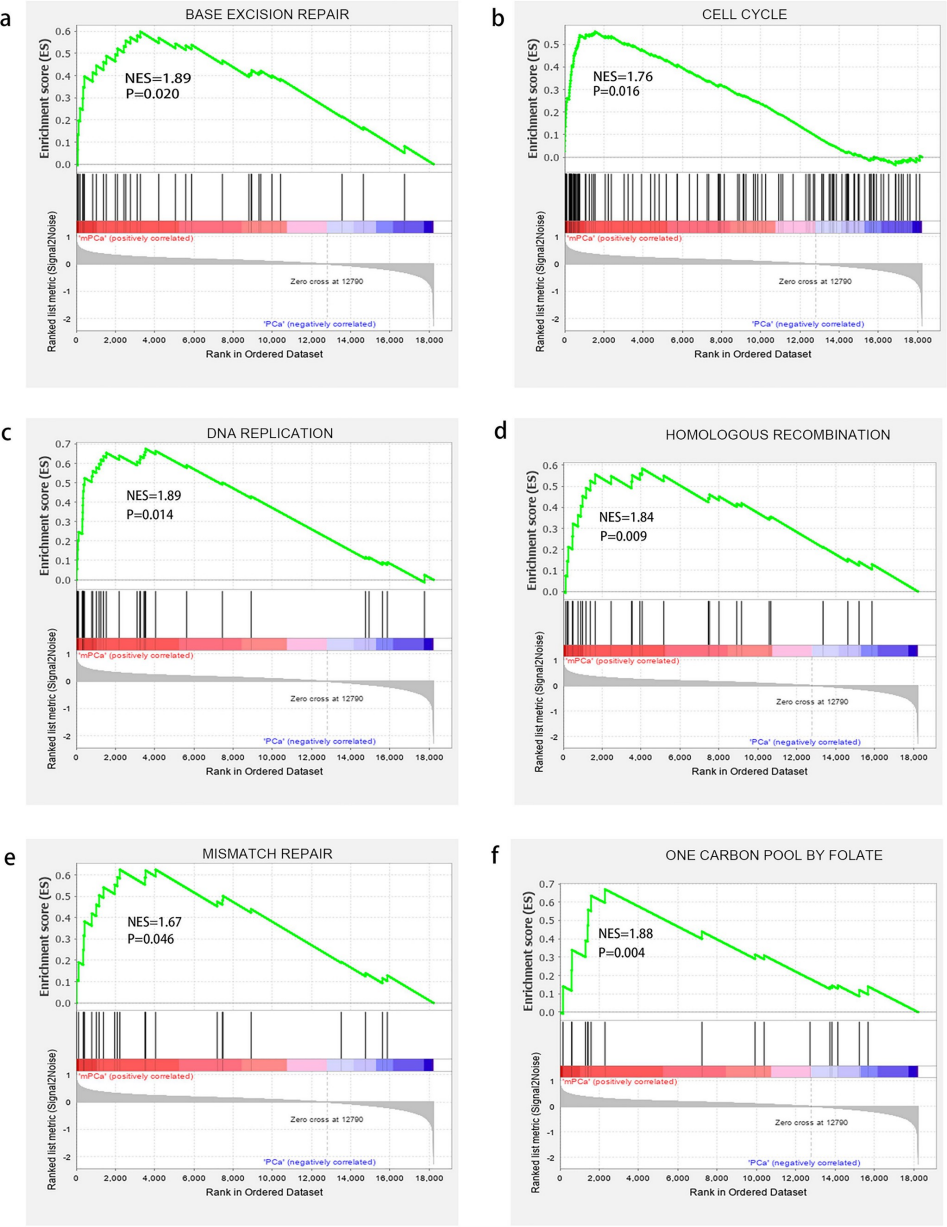
KEGG-GSEA was applied for the signaling pathway analysis. Pathways: A. base excision repair; B. cell cycle; C. DNA replication; D. homologous recombination; E. mismatch repair; F. one carbon pool by folate.

To probe the biological process of target mRNAs, ClusterProfiler packages of R were used to perform GO analysis (Table 4). We found that the upregulated mRNAs were related to the interleukin-7-mediated signaling pathway, chromatin silencing at rDNA, cellular response to interleukin-7 and protein heterooligomerization. In addition, we found that the base excision repair, cell cycle, one carbon pool by folate, DNA replication, mismatch repair and homologous recombination were related to genes that were upregulated in the mPCa samples by KEGG-GSEA (Fig 6).

**Table 4:** GO analysis for differential mRNAs in the competing endogenous RNA network.

Categories	Terms	Description	P-adjust	Count
BP	GO:0038111	interleukin-7-mediated signaling pathway	6.67E-09	6
	GO:0000183	chromatin silencing at rDNA	1.25E-08	6
	GO:0098760	response to interleukin-7	1.25E-08	6
	GO:0098761	cellular response to interleukin-7	1.25E-08	6
	GO:0051291	protein heterooligomerization	2.65E-07	7
CC	GO:0000786	nucleosome	1.83E-06	6
	GO:0044815	DNA packaging complex	1.83E-06	6
	GO:0032993	protein-DNA complex	2.42E-05	6
	GO:0071682	endocytic vesicle lumen	0.000136565	3
	GO:0000785	chromatin	0.000380551	7
MF	GO:0046982	protein heterodimerization activity	0.002416344	7
	GO:0004601	peroxidase activity	0.002416344	3
	GO:0016684	oxidoreductase activity, acting on peroxide as acceptor	0.002416344	3
	GO:0016209	antioxidant activity	0.007751172	3

Go, Gene ontology.

### Survival analysis of hub genes

**Fig 7:**
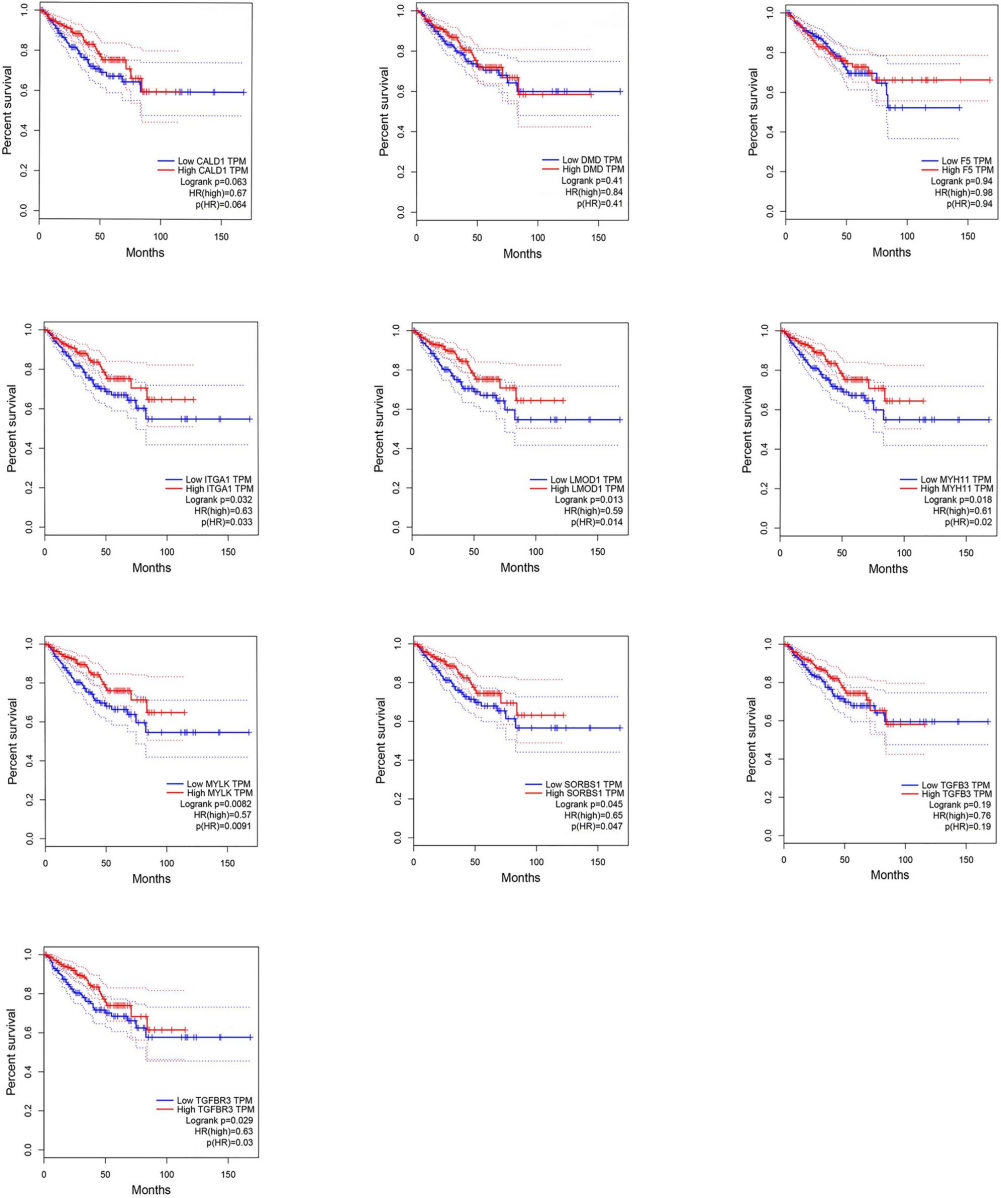
Disease-free survival plot of 10 hub genes.

We analyzed the DFS of the 10 genes by GEPIA with a cutoff value of p <0.05. The results indicated that the expression levels of ITGA1, LMOD1, MYH11, MYLK, SORBS1 and TGFBR3 were positively associated with DFS in patients with PCa (Fig 7).

## Discussion

Recently, due to the high attention given to the ceRNA hypothesis, the development of the ceRNA network has been very fast and contributed to a deep perception of the function and mechanism of ceRNAs. Increasing evidence has shown that ceRNAs play remarkable roles in tumor development and advancement by changing the expression of pivotal oncogenic or tumor-suppressive genes [10–12]. Wang et al. proposed a ceRNA network comprised of 32 lncRNAs, 14 miRNAs and 158 mRNAs in Wilms tumor (WT) based on the expression patterns of TARGET and GEO databases to illuminate the mechanism in WT pathogenesis [13]. Liu et al. identified 6 circRNAs and 6 miRNAs and 36 mRNAs to analyze and find possible pathogenesis and potential treatments of stomach adenocarcinoma [14]. In the current study, we identified 45 circRNAs, 24 miRNAs and 56 mRNAs to build a ceRNA network that could shed new light on the occurrence and development of mPCa.

Certain circRNAs in the ceRNA network can act as effective clinical biomarkers because they are related to cancer cell growth, progression and metastasis. For example, a study indicated that hsa_circ_0022426 could inhibit triple-negative breast proliferation, migration and invasion [15]. Yu et al. reported that knockdown of hsa_circ_0059780 could inhibit non-small-cell lung carcinoma growth by regulating miR-206 [16]. Hsa_circ_0085539 was connected with osteosarcoma progression by sponging miR-526b-5p and suppressing SERP1 [17]. Hsa_circ_0001460 was shown to regulate the expression of ADAR1 by sponging miR-432-5p, influencing the cell cycle progression and promoting the epithelial-to-mesenchymal transition in pancreatic ductal adenocarcinoma cells [18]. Li et al. indicated that hsa_circ_0001460 participated in the partial development of hepatocellular carcinoma by modulating miR-3150b-3p/LAMC1 [19]. However, the details of circRNAs in mPCa are not entirely illustrated. Our research aimed to explore a novel method to promote the comprehensive recognition of the underlying molecular mechanism of circRNAs in mPCa by identifying the relationships of circRNAs, miRNAs and mRNAs.

Increasing evidence has proven the important effect of miRNAs in cancer cell proliferation, metastasis and immune evasion, which can be used as possible diagnostic and therapeutic methods in various cancers [20–22]. In the present research, we found 24 DEmiRNAs in the ceRNA network. Certain studies have demonstrated that certain DEmiRNAs are necessary for tumor development. MiR-296-5p is considered a valuable miRNA, and a report showed that the overexpression of miR-296-5p restricted the tumor development and aggression of hepatocellular carcinoma (HCC) by inactivating NRG1-Fra-2 signaling [23]. In colorectal cancer (CRC), cellular experiments have proven that downregulated miR-548c-5p restrains cancer cell growth and reproduction of inflammatory cytokines by directly targeting PGK1 [24]. Downregulation of miR-548c-5p could predict poor survival in CRC [25]. Similarly, miR-665 could predict the poor survival and facilitate the metastasis of breast cancer via the NR4A3-MEK signaling pathway [26]. CircABCC2 has the potential to bind miR-665 to manage ABCC2 expression, which affects the development and progression of HCC [27]. Thus, miRNAs play an essential role in cancer progression, and the 24 DEmiRNAs involved in the ceRNA network may have inseparable relations with mPCa.

Among the 56 DEmRNAs in the ceRNA regulatory network, multivariable analysis indicated that the MEIS2 expression correlated with a reduction in the metastatic progression of PCa [28]. Yan et al. demonstrated that CNTN1, which is a type of cell adhesion protein in the neural system, could promote the PCa cell invasion, play a part in tumor formation and enhance metastasis [29]. The oncoprotein STMN1 could boost the PCa cell growth and aggression. miR-34a downregulated STMN1 to inhibit cancer proliferation and colony formation [30]. DDR2 in PCa may stimulate bone resorption by up-regulating RANKL, and promote the adhesion between cancer cells and type I collagen, which could induce bone metastasis [31]. To further investigate the association between DEmRNAs and clinical prognosis, GEPIA was used. A panel of 6 hub genes (ITGA1, LMOD1, MYH11, MYLK, SORBS1 and TGFBR3) significantly correlated with DFS in patients with PCa. Among the 6 hub genes, LMOD1, MYLK, SORBS1 and TGFBR3 were demonstrated to be important in the tumorigenesis and metastasis of PCa. Kawahara et al. used parallel reaction monitoring to prove that as a member of a protein pane, LMOD1, which can differentiate low and high PCa grade samples, might be a biomarker for the development of PCa [32]. Researchers identified that circRNA-MYLK was significantly expressed in PCa tissues and could regulate the miR-29a expression levels to promote the progression of PCa [33]. Anna et al. reported that SORBS1 was negatively associated with its supposed miRNAs. When SORBS1 was downregulated, patients with PCa were inclined to biochemical relapse [34]. Previous research indicated that the expression of TGFBR3 in primary PCa was obviously higher than that in mPCa samples, and lower TGFBR3 was related to poor clinical outcome in patients with PCa. The TGFβRIII-p38MAPK-pS249/pT252-RB signaling pathway participates in dormant induction in PCa cells and could provide a novel route for researchers to develop methods that may prevent dormant tumors from appearing in PCa patients [35].

There are some limitations in this study. Firstly, we only investigated the circular RNA-mediated regulatory network and ignored other regulatory models such as lncRNAs. Secondly, bioinformatic methods were used to explore ceRNAs associated with mPCa, and further validation studies using clinical samples must be conducted in the future.

## Conclusion

From publicly available databases, we found 45 DEcircRNAs, 24 DEmiRNAs, and 56 DEmRNAs and established a circRNA-mediated ceRNA regulatory network, which can provide potential biomarkers for the prognosis of mPCa. The fundamental mechanism of these ceRNAs should be further explored.
